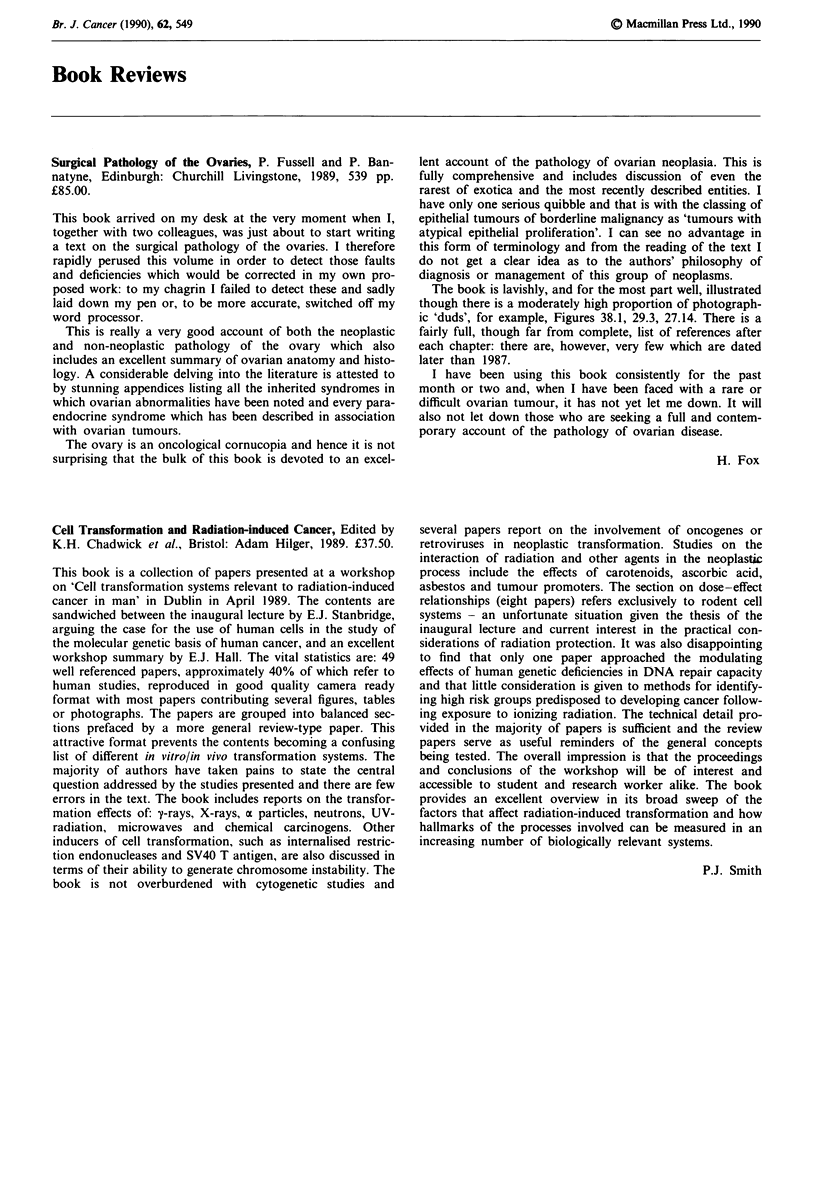# Surgical Pathology of the Ovaries

**Published:** 1990-09

**Authors:** H. Fox


 © Macmillan Press Ltd., 1990

### Book Reviews

**Surgical Pathology of the Ovaries,** P. Fussell and P. Bannatyne, Edinburgh: Churchill Livingstone, 1989, 539 pp. £85.00.

This book arrived on my desk at the very moment when I, together with two colleagues, was just about to start writing a text on the surgical pathology of the ovaries. I therefore rapidly perused this volume in order to detect those faults and deficiencies which would be corrected in my own proposed work: to my chagrin I failed to detect these and sadly laid down my pen or, to be more accurate, switched off my word processor.

This is really a very good account of both the neoplastic and non-neoplastic pathology of the ovary which also includes an excellent summary of ovarian anatomy and histology. A considerable delving into the literature is attested to by stunning appendices listing all the inherited syndromes in which ovarian abnormalities have been noted and every paraendocrine syndrome which has been described in association with ovarian tumours.

The ovary is an oncological cornucopia and hence it is not surprising that the bulk of this book is devoted to an excellent account of the pathology of ovarian neoplasia. This is fully comprehensive and includes discussion of even the rarest of exotica and the most recently described entities. I have only one serious quibble and that is with the classing of epithelial tumours of borderline malignancy as 'tumours with atypical epithelial proliferation'. I can see no advantage in this form of terminology and from the reading of the text I do not get a clear idea as to the authors' philosophy of diagnosis or management of this group of neoplasms.

The book is lavishly, and for the most part well, illustrated though there is a moderately high proportion of photographic 'duds', for example, Figures 38.1, 29.3, 27.14. There is a fairly full, though far from complete, list of references after each chapter: there are, however, very few which are dated later than 1987.

I have been using this book consistently for the past month or two and, when I have been faced with a rare or difficult ovarian tumour, it has not yet let me down. It will also not let down those who are seeking a full and contemporary account of the pathology of ovarian disease.

H. Fox